# Supplementary material for: Disrupting Plasmodium UIS3–host LC3 interaction with a small molecule causes parasite elimination from host cells
Source: Commun Biol. 2020 Nov 19;3:688. doi: 10.1038/s42003-020-01422-1 (PMC7677311; doi:10.1038/s42003-020-01422-1)
Supplement: Supplementary file 1 — Supplementary Information [file 42003_2020_1422_MOESM1_ESM.pdf]

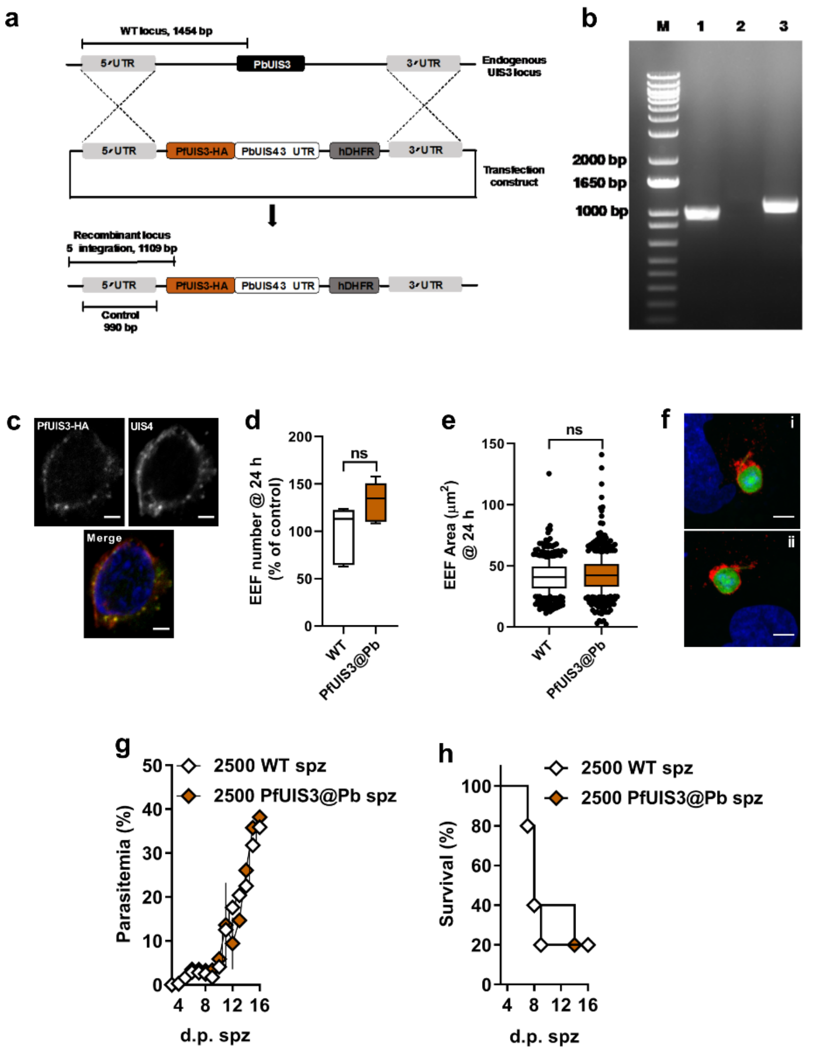

Supplementary Figure 1. Generation of PfUIS3@Pb parasites. (**a**) Schematic representation of the approach used for replacing *uis3* gene of *P. berghei* ANKA (507cl1) with that of *P. falciparum* 3D7, marked with 2 copies of HA tag. The location of the different primers pairs used to verify recombination by PCR and the expected length of the amplicons are indicated. (**b**) PCR confirming integration of PfUIS3-HA replacement fragment using genomic DNA from PfUIS3@Pb parasites. The primers used are listed in Supplementary Table 2 and 3. 1: Control (5` UTR of *Pbuis3* gene); 2: WT locus; 3: PfUIS3-HA recombinant locus 5` integration. (**c**) PVM localization of PfUIS3-HA. Representative confocal images (n=2 independent experiments) of HuH7 cells infected with PfUIS3@Pb parasites. 24 post-infection cells were fixed and immunostained with anti-HA (green), anti-UIS4 (red) and Hoechst (blue). Scale bars = 2 µm. (**d and e**) Comparison of the EEFs number (**d**) and EEFs size (**e**) of WT and PfUIS3@Pb parasite at 24 h post-infection. Here, data is represented as box-plot (10-90 percentile), n=2 independent experiments. N ≥ 100 parasites. Statistical significance was assessed using the non-parametric two-tailed Mann-Whitney test. ns: non-significant. (**f**) Representative confocal images of EEFs size comparison (i: WT, ii: PfUIS3@Pb). Cells were stained with anti-UIS4 (red), anti-GFP (green) and Hoechst (blue). Scale bars = 5 µm. (**g and h**) Comparison of the blood parasitaemia (**g**) and survival of the mice (**h**) infected with 2500 sporozoites from WT or PfUIS3@Pb parasites. Data represent mean (of 5 mice in each group) ± SEM. d.p.spz: day post-sporozoite.

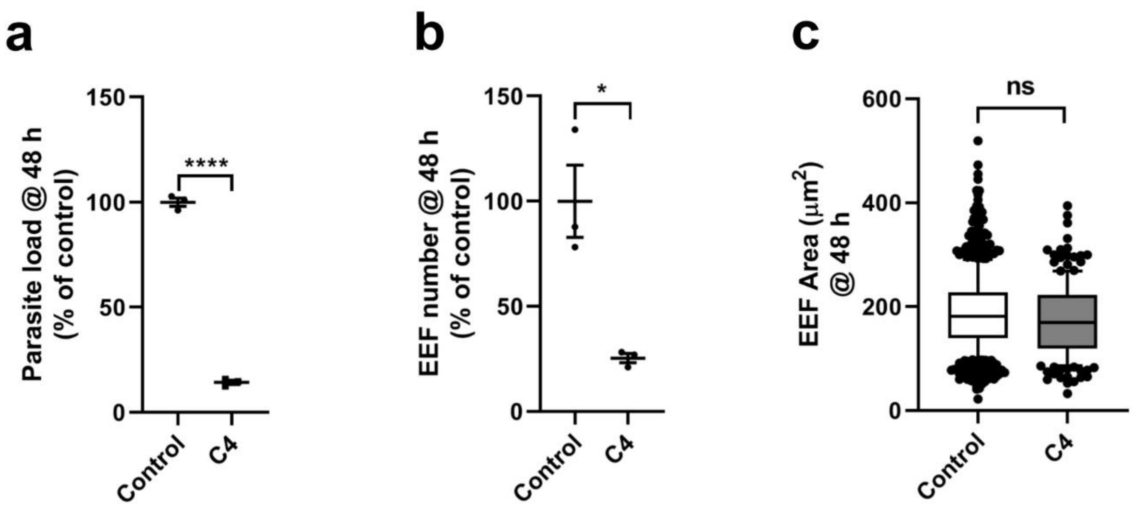

Supplementary Figure 2. Effect of C4 treatment on *Plasmodium* development at 48 h post-infection. Luminescence and microscopy based quantification of the effect of 1 μM C4 treatment on parasite load (**a**), EEFs number (**b**), and EEFs size (mean GFP intensity, **c**) in *P. berghei* infected Huh7 cells. In (**a and b**) data represents means ± SEM. In (**c**) data is represented as box-plot (10-90 percentile). In (**a-c**) n=1. In (**b and c**) N ≥ 100 parasites. Statistical significance was assessed using unpaired two-tailed *t*-test. ns: non-significant, * $P<0.1$, **** $P<0.0001$.

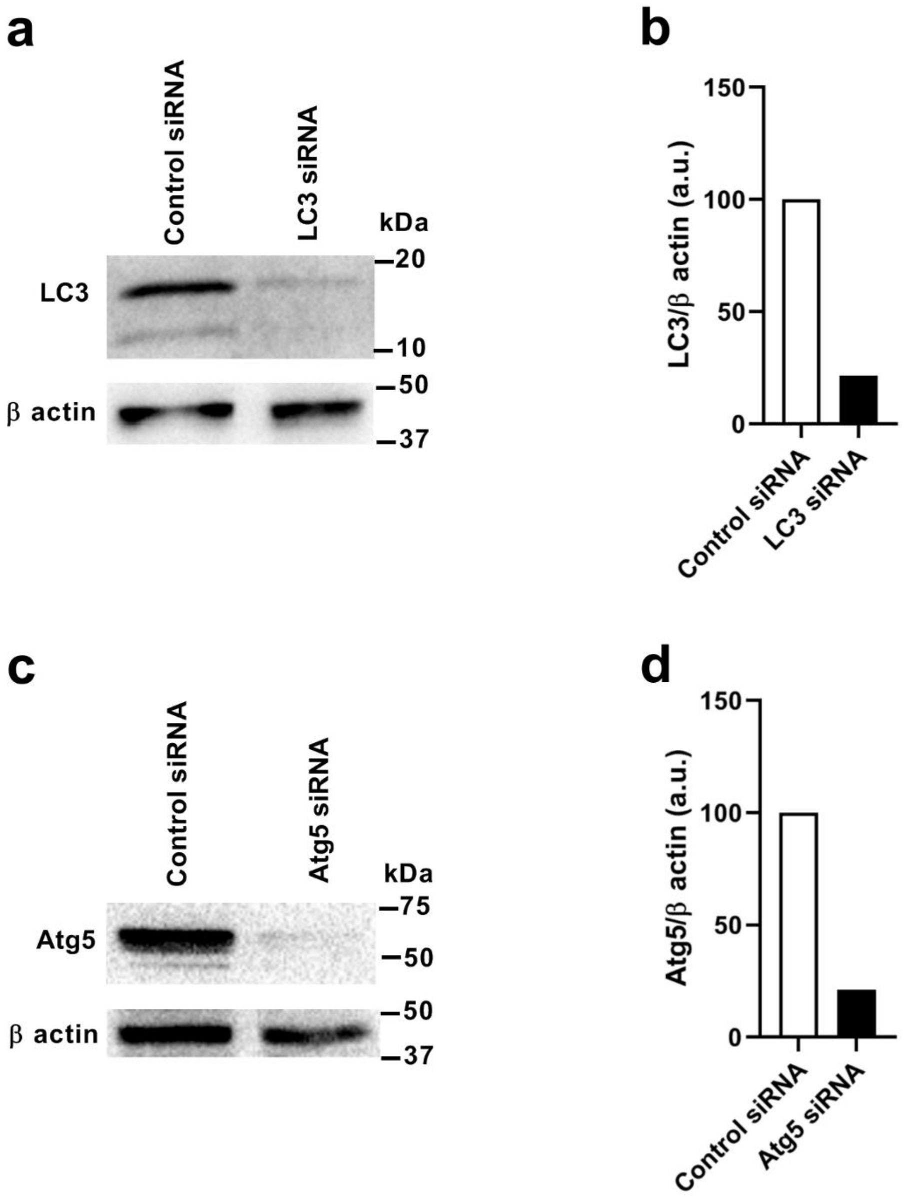

Supplementary Figure 3. Optimization of LC3 and Atg5 knockdown in HeLa cells. (**a and b**) Expression of LC3 in HeLa cells 24 h after siRNA transfection. (**c and d**) Expression of Atg5 in HeLa cells 48 h after siRNA transfection. For (**a-d**) n=2 independent experiments. One representative experiment is shown in each case. (**a and c**) Representative immunoblots. (**b and d**) Quantification of the target gene expression after siRNA transfection. β actin – loading control.

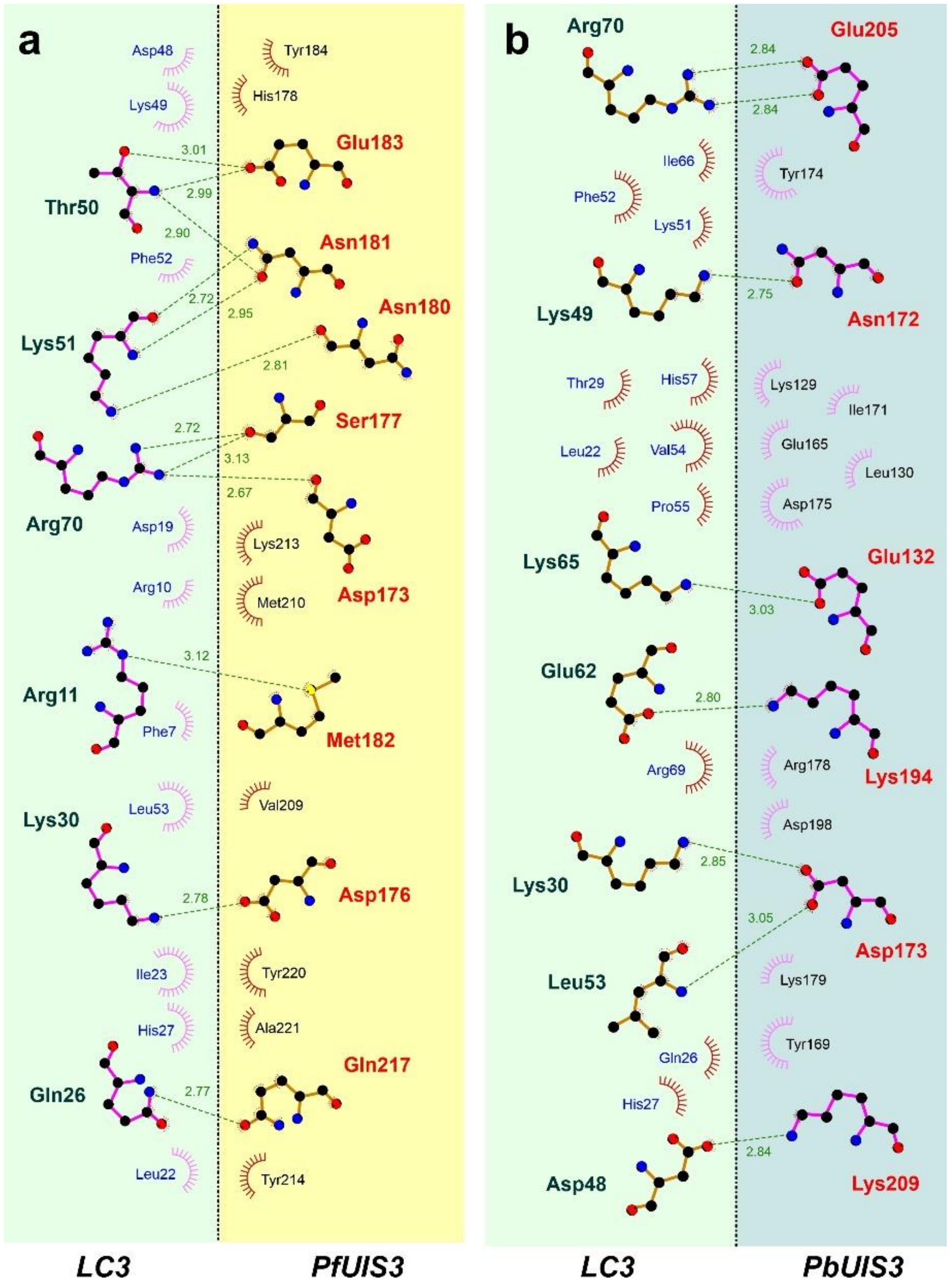

Supplementary Figure 4. Two dimensional representation of all the interactions involved in the predicted UIS3-LC3 complex in *P. falciparum* (a) and *P.berghei* (b). The green dotted lines represent the hydrogen bond formation between LC3 and UIS3 residues, showing the inter-atomic distances in Amgstrons. Hydrophobic interactions between neighbour residues are depicted by arcs following the Ligplot+ convention[1].

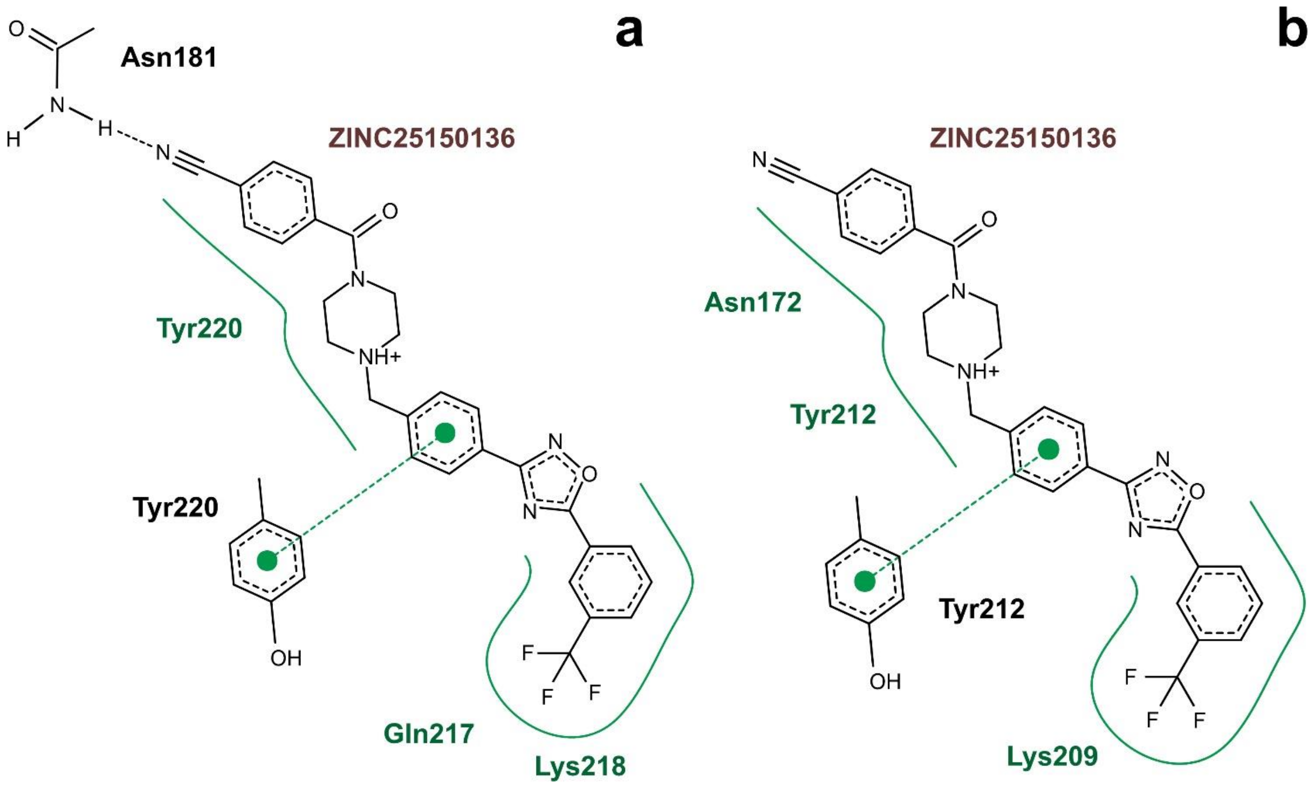

Supplementary Figure 5. Two-dimensional plot of compound C4 and the predicted interactions with PfUIS3 (a) and PbUIS3 (b). Green continuous lines represent the hydrophobic interactions between residues and ZINC25150136 compound. The pi-mediated interactions between Tyr220 in *P. falciparum* and Tyr212 in *P. berghei* are depicted as slashed lines. Figure produced by Poseview software[2].

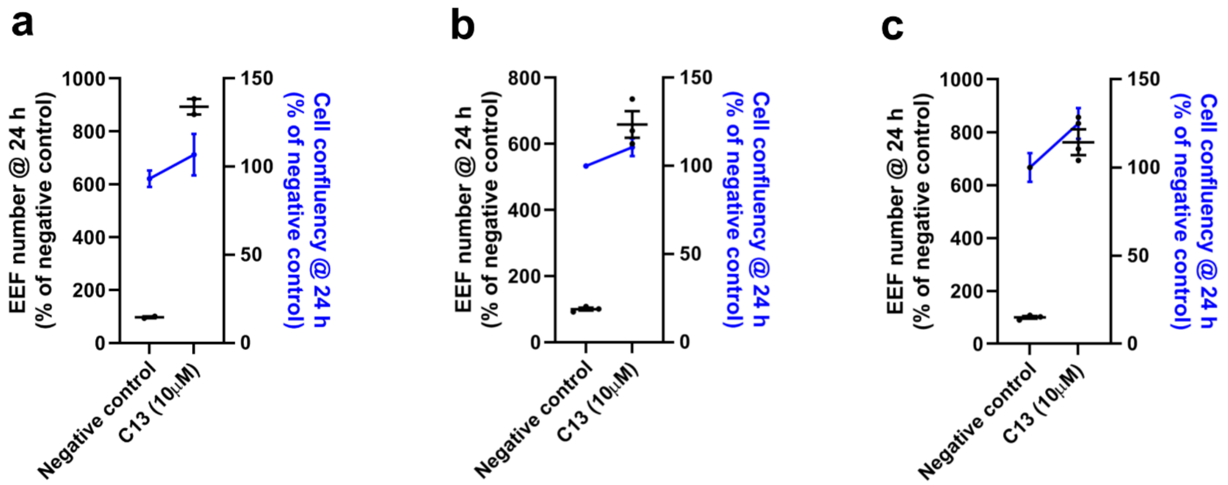

Supplementary Figure 6. Effect of C13 treatment on PfUIS3@Pb infected Huh7 cells at 24 h post-infection. Individual data of three independent experiments are presented. Here, scatter plots represent the number of EEFs, as quantified by the number of infected cells using flow cytometry. The blue lines represent cell confluency. Data represent means ± SEM.

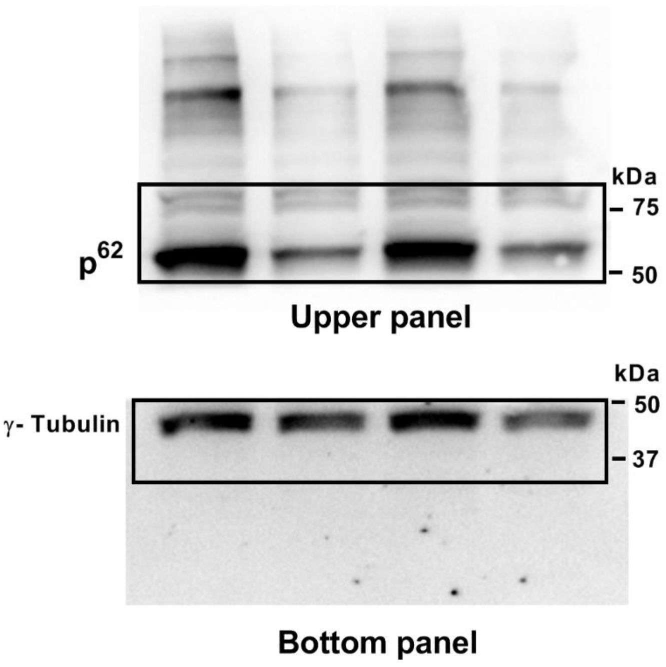

Supplementary Figure 7. Raw data of Figure 6. The boxes show where the images were cropped to produce the main figure.

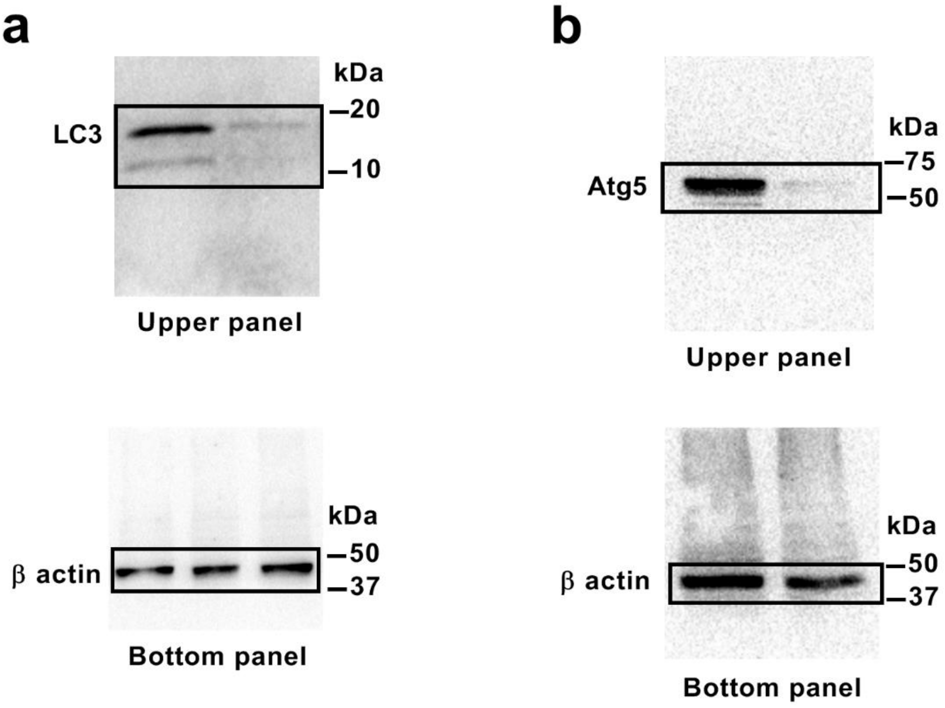

Supplementary Figure 8. Raw data of Supplementary Figure 3. The boxes show where the images were cropped to produce the supplementary figure.

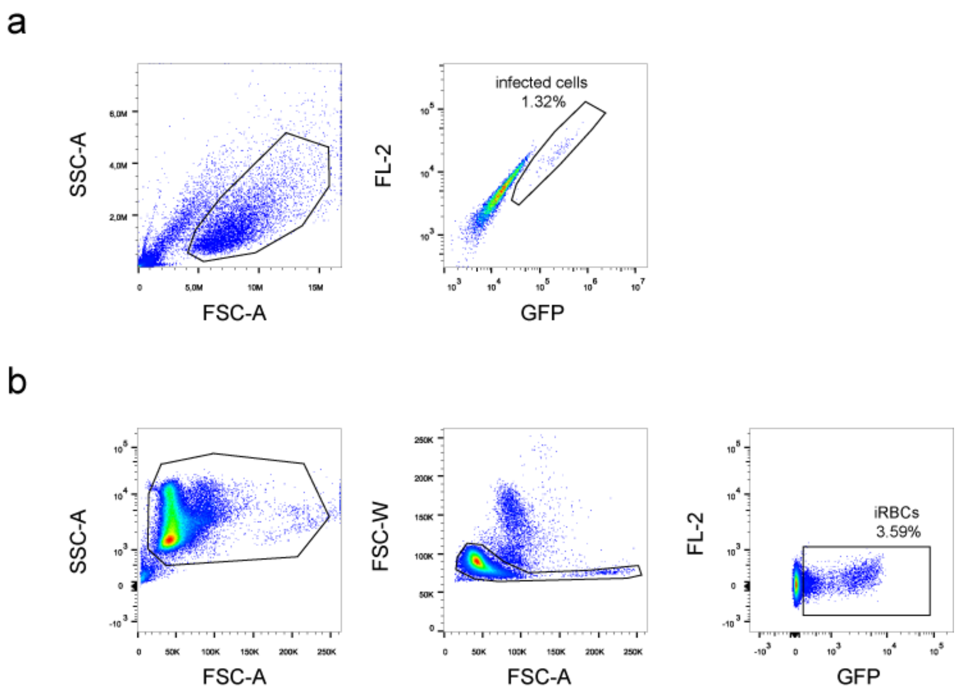

Supplementary Figure 9. Flow cytometry gating strategy. (a) Gating strategy for infected Huh7 or HeLa to quantify the number of EEFs. **(b)** Gating strategy for infected RBC (iRBC) to quantify parasitaemia in mice.

Supplementary Table 1. Compounds used in this study

Compound	ZINC ID	Structure	Molecular Weight (g/mol)	xlogP	Rotatable Bonds	Polar surface area (Å^2)
C1	15243482	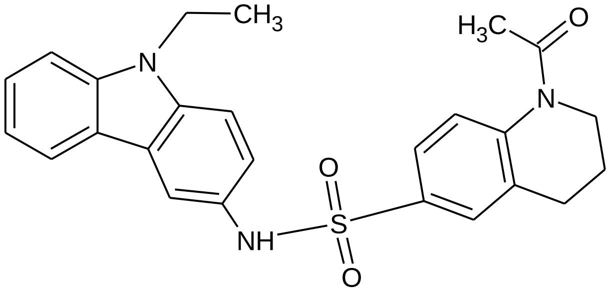	447.56	4.60	4	71
C2	873264	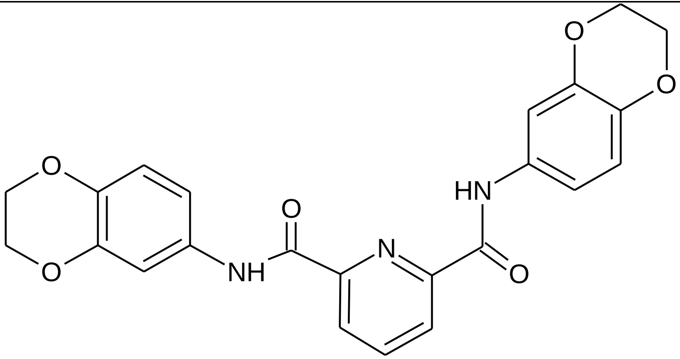	433.42	1.64	4	108
C3	7599237	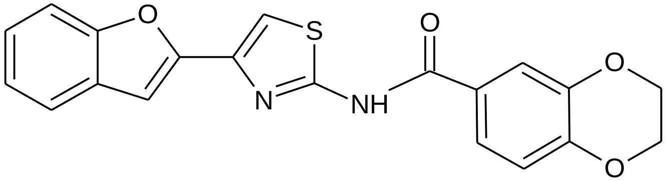	378.40	3.51	3	73
C4	25150136	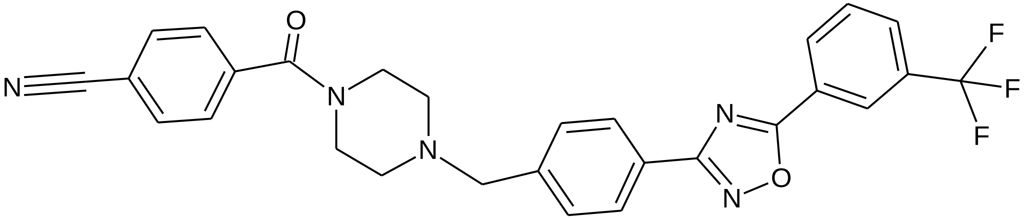	517.51	4.61	6	86

C5	14242199	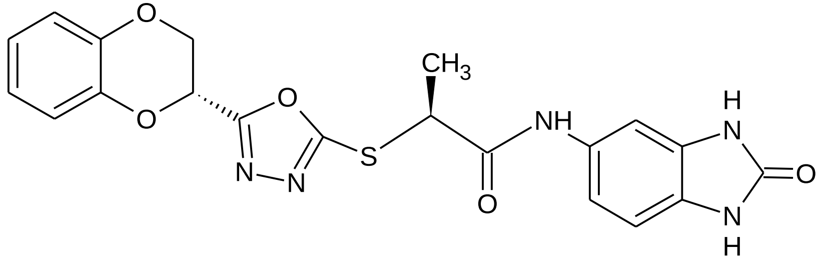	439.45	1.56	5	135
C6	35399629	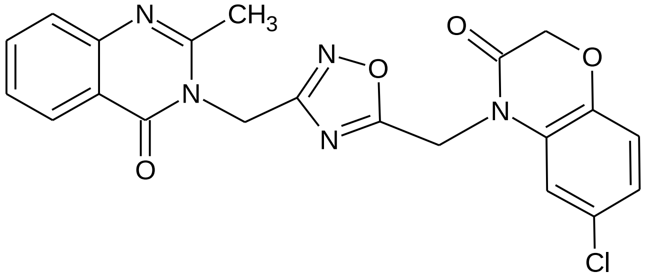	437.84	2.18	4	103
C7	9419593	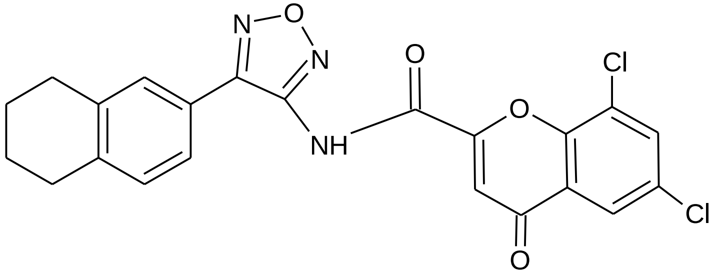	456.28	5.65	3	98
C8	36076978	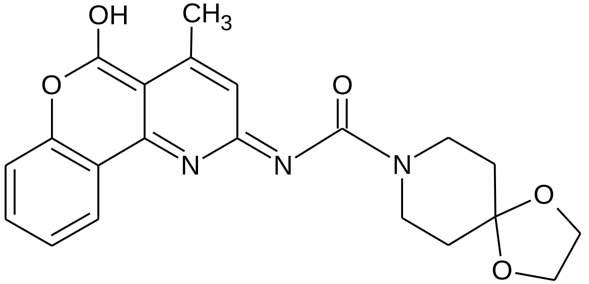	411.45	3.07	2	100

C9	32999120	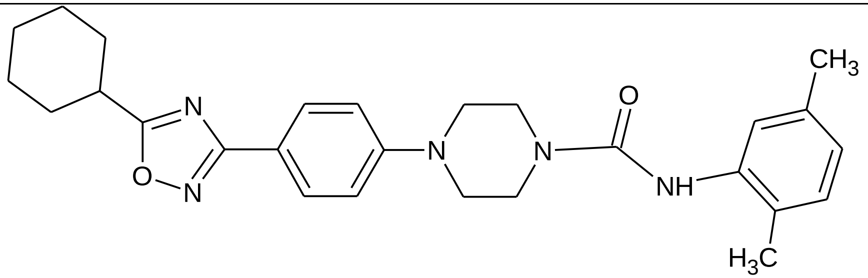	459.59	6.10	4	74
C10	10994401	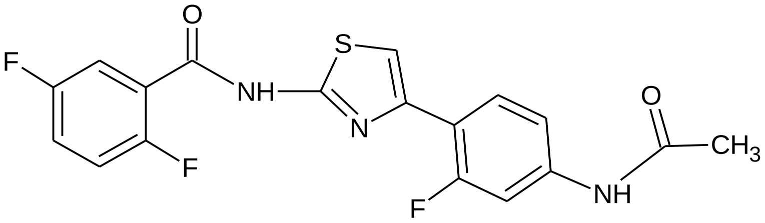	391.37	3.12	4	71
C11	72128248	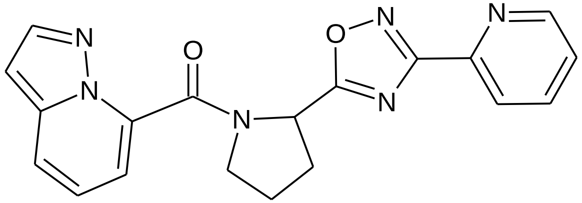	360.37	1.74	3	89
C12	20610177	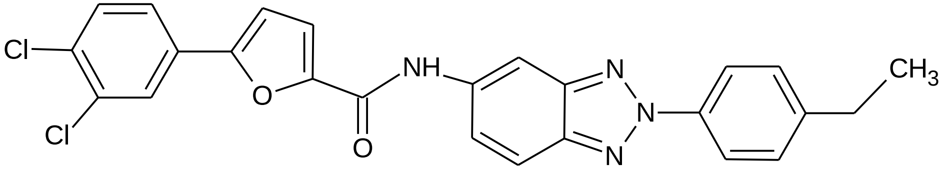	477.35	6.87	5	73
C13	19852631	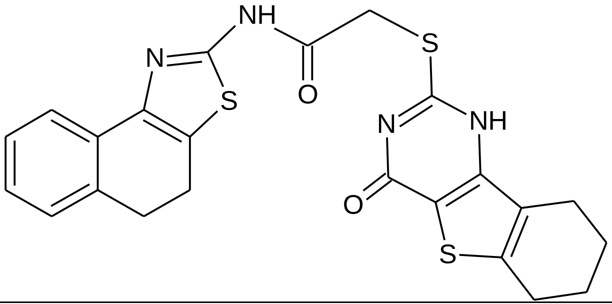	480.64	5.34	4	88

C14	66982809	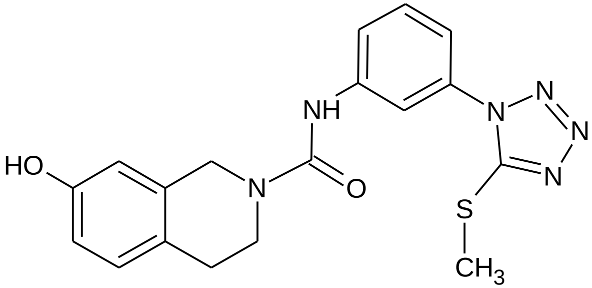	382.44	2.34	3	96
C15	64193563	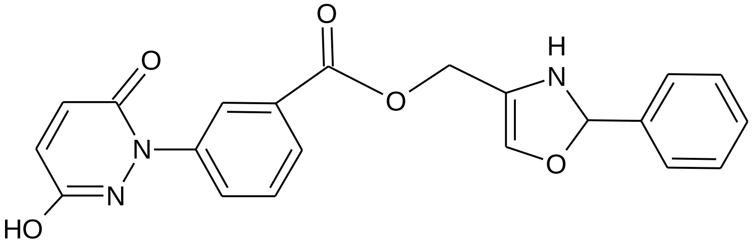	388.35	3.72	6	110

Note: Compound 8 and 9 were excluded from the screening due to insolubility in DMSO.

Supplementary Table 2. Primers used to generate the transfection construct

PCR reaction	Primer pairs (Forward; Reverse)
PfUIS3-HA	5´ ATGGGCCCATGAAGGTCTCTAAATTAGTCTTG 3´;<br><br>5´ATGCGGCCGCTTATGCATAATCTGGTACATCATATGGA TATGCATAATCTGGTACATCATATGGATAGTTCTCTTCTT GAGATAAATAATTAG 3´
PbUIS3 5´ UTR	5´ TAAAGCTTAAGGATTATATTTTATAATGTTTCAC 3´;<br><br>5´ ATGGGCCCTTTTATACACTTTCATATATTTGTTAT 3´
PbUIS3 3´ UTR	5´ TAGGTACCATGTTTGTGTAACATCATTTATAG 3´;<br><br>5´ ATAAGCTTTTCATATATCAATTTTCAAATTG 3´
PbUIS4 3´ UTR	5´ ATGCGGCCGCTTCATTATGAGTAGTGTAATTCAG 3´;<br><br>5´ ATGAATTCGCATACAACATATGTAAAAAAG 3´

Supplementary Table 3. Primers used for PfUIS3@Pb genotyping

PCR reaction	Primer pairs (Forward; Reverse)
Recombinant locus 5´ integration	5´ ATTATATTGTGCTATAAAGCG 3´<br><br>5´ GCATTTAAACAAATATATTGACATAAGAT 3´
WT locus	5´ TAAAGCTTAAGGATTATATTTTATAATGTTTCAC 3´<br><br>5´ GCAGCTAGTTTCACATTATCCATAAATAT 3´
Control (PbUIS3 5´ UTR)	5´ TAAAGCTTAAGGATTATATTTTATAATGTTTCAC 3´;<br><br>5´ ATGGGCCCTTTTATACACTTTCATATATTTGTTAT 3´

Supplementary References

1. Laskowski, R. A. & Swindells, M. B. LigPlot+: multiple ligand-protein interaction diagrams for drug discovery. *J. Chem. Inf. Modeling* **51**, 2778–2786 (2011).

2. Fahrrolfes, R. et al. ProteinsPlus: a web portal for structure analysis of macromolecules. *Nucleic Acids Res.* **45**, W337–W343 (2017).
