# Supplementary material for: Disrupting Plasmodium UIS3–host LC3 interaction with a small molecule causes parasite elimination from host cells
Source: Commun Biol. 2020 Nov 19;3:688. doi: 10.1038/s42003-020-01422-1 (PMC7677311; doi:10.1038/s42003-020-01422-1)
Supplement: Supplementary file 3 — Description of Additional Supplementary Files [file 42003_2020_1422_MOESM3_ESM.pdf]

Description of Additional Supplementary Files

File Name: Supplementary Data 1
Description: Source data for main figures.

File Name: Supplementary Data 2
Description: Chemdraw file for C4 structure

File Name: Supplementary Data 3
Description: Details of the statistical analysis performed during this study
