# Supplementary material for: Disrupting Plasmodium UIS3–host LC3 interaction with a small molecule causes parasite elimination from host cells
Source: Commun Biol. 2020 Nov 19;3:688. doi: 10.1038/s42003-020-01422-1 (PMC7677311; doi:10.1038/s42003-020-01422-1)
Supplement: Supplementary file 7 — Reporting Summary [file 42003_2020_1422_MOESM7_ESM.pdf]

nature research

Corresponding author(s): Maria Manuel Mota

Last updated by author(s): Oct 13, 2020

Reporting Summary

Nature Research wishes to improve the reproducibility of the work that we publish. This form provides structure for consistency and transparency in reporting. For further information on Nature Research policies, see our Editorial Policies and the Editorial Policy Checklist.

Statistics

For all statistical analyses, confirm that the following items are present in the figure legend, table legend, main text, or Methods section.

n/a	Confirmed	
☐	☒	The exact sample size (*n*) for each experimental group/condition, given as a discrete number and unit of measurement
☐	☒	A statement on whether measurements were taken from distinct samples or whether the same sample was measured repeatedly
☐	☒	The statistical test(s) used AND whether they are one- or two-sided<br>*Only common tests should be described solely by name; describe more complex techniques in the Methods section.*
☒	☐	A description of all covariates tested
☒	☐	A description of any assumptions or corrections, such as tests of normality and adjustment for multiple comparisons
☐	☒	A full description of the statistical parameters including central tendency (e.g. means) or other basic estimates (e.g. regression coefficient) AND variation (e.g. standard deviation) or associated estimates of uncertainty (e.g. confidence intervals)
☐	☒	For null hypothesis testing, the test statistic (e.g. *F*, *t*, *r*) with confidence intervals, effect sizes, degrees of freedom and *P* value noted<br>*Give P values as exact values whenever suitable.*
☒	☐	For Bayesian analysis, information on the choice of priors and Markov chain Monte Carlo settings
☒	☐	For hierarchical and complex designs, identification of the appropriate level for tests and full reporting of outcomes
☒	☐	Estimates of effect sizes (e.g. Cohen's *d*, Pearson's *r*), indicating how they were calculated

Our web collection on statistics for biologists contains articles on many of the points above.

Software and code

Policy information about availability of computer code

Data collection	No software was used.
Data analysis	Data analysis softwares : Flow cytometry (FlowJo), Immunofluorencence (ImageJ), WB quantification (Image Lab). All statistical tests were performed in GraphPad Prism.

For manuscripts utilizing custom algorithms or software that are central to the research but not yet described in published literature, software must be made available to editors and reviewers. We strongly encourage code deposition in a community repository (e.g. GitHub). See the Nature Research guidelines for submitting code & software for further information.

Data

Policy information about availability of data

All manuscripts must include a data availability statement. This statement should provide the following information, where applicable:

- Accession codes, unique identifiers, or web links for publicly available datasets
- A list of figures that have associated raw data
- A description of any restrictions on data availability

All data generated or analysed during this study are included in this manuscript and its Supplementary Information.

Field-specific reporting

Life sciences study design

All studies must disclose on these points even when the disclosure is negative.

Sample size	Sample sizes on mice experiments were chosen on the basis of historical data.
Data exclusions	No data were excluded.
Replication	Authors have mentioned the number of replication for all experiments in respective Figure legends and Supplementary Data 3. All attempts were successful.
Randomization	Radomization was not relevant to this study.
Blinding	To study Lamp1 dynamics around the PVM after C4 treatment, samples were blinded before imaging and image analysis.

Reporting for specific materials, systems and methods

We require information from authors about some types of materials, experimental systems and methods used in many studies. Here, indicate whether each material, system or method listed is relevant to your study. If you are not sure if a list item applies to your research, read the appropriate section before selecting a response.

Materials & experimental systems

n/a	Involved in the study
☐	☒ Antibodies
☐	☒ Eukaryotic cell lines
☒	☐ Palaeontology and archaeology
☐	☒ Animals and other organisms
☒	☐ Human research participants
☒	☐ Clinical data
☒	☐ Dual use research of concern

Methods

n/a	Involved in the study
☒	☐ ChIP-seq
☐	☒ Flow cytometry
☒	☐ MRI-based neuroimaging

Antibodies

Antibodies used

Immunofluorescence primary antibodies: PbUIS4 (goat polyclonal, SicGen, AB0042-200, 1:1000), HA (mouse monoclonal, BioLegend, 901509, 1:500) and Lamp1 (rabbit polyclonal, Sigma, L1418, 1:1000). GFP signal was detected using AlexaFluor 488 conjugated anti GFP (rabbit polyclonal, Invitrogen, A-21311, 1:500) antibody. AlexaFluor-conjugated immunofluorescence secondary antibodies were from Invitrogen.
Western Blot antibodies: p62 (rabbit polyclonal, Sigma-Aldrich, P0067, 1:1000), gamma-tubulin (mouse monoclonal, Sigma-Aldrich, T5326, 1: 10,000), LC3 (rabbit polyclonal, MBL, PM036, 1:1000), Atg5 (rabbit polyclonal, Cell Signalling Technology, 2630, 1:1000) and β actin (mouse monoclonal, Abcam, ab8224, 1:1000).

Validation

Validation statement of each antibody can be found on the manufacturer`s website.

Immunofluorescence primary antobodies
PbUIS4: http://www.sicgen.pt/product/uis4-polyclonal-antibody_1_11
HA: https://www.biolegend.com/en-us/products/alexa-fluor-488-anti-ha-11-epitope-tag-antibody-10993
Lamp1:https://www.sigmaaldrich.com/catalog/product/sigma/l1418?lang=pt&region=PT
GFP: https://www.thermofisher.com/antibody/product/GFP-Antibody-Polyclonal/A-21311

Immunofluorescence secondary antobodies
https://www.thermofisher.com/antibody/product/Donkey-anti-Goat-IgG-H-L-Cross-Adsorbed-Secondary-Antibody-Polyclonal/A-11057
https://www.thermofisher.com/antibody/product/Goat-anti-Mouse-IgG-H-L-Highly-Cross-Adsorbed-Secondary-Antibody-Polyclonal/A-21236
https://www.thermofisher.com/antibody/product/Donkey-anti-Goat-IgG-H-L-Cross-Adsorbed-Secondary-Antibody-Polyclonal/A-11055
https://www.thermofisher.com/antibody/product/Donkey-anti-Rabbit-IgG-H-L-Highly-Cross-Adsorbed-Secondary-Antibody-Polyclonal/A-31573

Western Blot primary antobodies
p62: https://www.sigmaaldrich.com/content/dam/sigma-aldrich/docs/Sigma/Datasheet/2/p0067dat.pdf
gamma-tubulin: https://www.sigmaaldrich.com/content/dam/sigma-aldrich/docs/Sigma/Datasheet/1/t5326dat.pdf
LC3:https://www.mblintl.com/products/pm036/
Atg5:https://www.cellsignal.com/products/primary-antibodies/atg5-antibody/2630
β actin:https://www.abcam.com/beta-actin-antibody-mabcam-8224-loading-control-ab8224.html

Eukaryotic cell lines

Policy information about cell lines

Cell line source(s)	Huh7 and HeLa cells were purchased from ATCC.
Authentication	None of the cell lines used were authenticated.
Mycoplasma contamination	All cell line tested negative for mycoplasma contamination.
Commonly misidentified lines (See ICLAC register)	No commonly misidentified cell lines were used in this study.

Animals and other organisms

Policy information about studies involving animals; ARRIVE guidelines recommended for reporting animal research

Laboratory animals	C57BL/6J males, of age between 6 and 8 weeks .
Wild animals	The study did not involve wild animals.
Field-collected samples	The study did not involve samples collected from the filed.
Ethics oversight	All the protocols were approved by the internal animal care committee of Instituto de Medicina Molecular João Lobo Antunes, Portugal and were performed according to national and European regulations.

Note that full information on the approval of the study protocol must also be provided in the manuscript.

Flow Cytometry

Plots

Confirm that:

- [x] The axis labels state the marker and fluorochrome used (e.g. CD4-FITC).
- [x] The axis scales are clearly visible. Include numbers along axes only for bottom left plot of group (a 'group' is an analysis of identical markers).
- [x] All plots are contour plots with outliers or pseudocolor plots.
- [x] A numerical value for number of cells or percentage (with statistics) is provided.

Methodology

Sample preparation	For infected Huh7/ HeLa, cells were trypsinized and resuspended in 300 µl of media. 50 µl of each sample were analysed. For infected RBC (iRBC), 5 µl blood from each mouse was diluted in 200 µl PBS, and was analysed by flow cytometry.
Instrument	BD Accuri C6 (Huh7/HeLa) and BD LSFortessa (iRBC).
Software	FlowJo
Cell population abundance	Not relevant to this study.
Gating strategy	For infected Huh7/HeLa, cells were first gated in FSC (A) vs SSC (A). Within this cell population, GFP positive infected cells were gated in FITC (GFP) vs FL2. For iRBC, staring cell population was gated in FSC (A) vs SSC (A). Next, single cell population were gated in FSC (A) vs FSC (W). Within this cell population, GFP positive iRBC were gated in FITC (GFP) vs FL2.

- [x] Tick this box to confirm that a figure exemplifying the gating strategy is provided in the Supplementary Information.
